# Estrogenic Effect of *Scoparia dulcis* (Linn) Extract in Mice Uterus and In Silico Molecular Docking Studies of Certain Compounds with Human Estrogen Receptors

**DOI:** 10.18502/jri.v21i4.4329

**Published:** 2020

**Authors:** Khamhee Wangsa, Indira Sarma, Purbajyoti Saikia, Dhanabalan Ananthakrishnan, Hirendra Nath Sarma, Devadasan Velmurugan

**Affiliations:** 1- Department of Zoology, Rajiv Gandhi University, Rono Hills, Itanagar, Arunachal Pradesh, India; 2- Centre of Advanced Study in Crystallography and Biophysics, University of Madras, Guindy Campus, Chennai, India

**Keywords:** Endometrium, Estrogen receptors, In silico, Menstrual disorders, Molecular docking, Phytoestrogen, *Scoparia dulcis* (Linn)

## Abstract

**Background:**

*Scoparia dulcis* Linn. is reported to be used by women of Assam and Arunachal Pradesh in northeast India for treating menstrual disorders. *Scoparia dulcis* contains compounds that bind with estrogen receptors (ERα and ERβ) evidenced by increased PCNA in endometrial epithelium.

**Methods:**

Crude extract was orally administered at the dose of 500 *mg/kg* body weight/day to the female mice (60–70 days old) in five different groups. Each group containing six females included: (I) cyclic control, (II) cyclic extract treated, (III) Ovariectomized (OVX)-vehicle treated (Control), (IV) OVX-E2 treated (V) OVX- extract treated. Extract was administered for eight days to the cyclic groups and three days to the OVX groups. PCNA was detected immunohistochemically in uterine tissues and signals were analyzed by Image J software (NIH, USA). Compounds were separated by GC-MS and identified using NIST. In silico molecular docking studies was performed with human estrogen receptors (ERα and ERβ). Molecular dynamics (MD) simulations of the best interacting compound was done using gromacs.

**Results:**

The results showed cell proliferation in the uterine endometrium evidenced by PCNA. Two phytocompounds, Octadecanoic acid and methyl stearate showed binding affinity with ERα and ERβ.

**Conclusion:**

*Scoparia dulcis* contains compounds having binding affinity with ERα and ERβ. The present study is the first report on compounds from *Scoparia dulcis* showing binding affinity with human estrogen receptors which may have biological effect on female reproduction.

## Introduction

*Scoparia dulcis* Linn is an herb available in the tropical and subtropical regions of the world. In India, the herb is known for its medicinal values (1, 2). Certain ethnic groups of northeast India use the stem and leaves of this herb to treat menstrual disorder and dysmenorrhea as well as to treat miscarriage. Following this background, it is hypothesized in our present study that extract of *Scoparia dulcis* contains compound(s) that bind to estrogen receptors (ER) and stimulate cellular proliferation agonistically. Estrogen is known for the proliferation of the uterine endometrium required for the proper implantation and development of the embryo (3, 4). Cellular proliferation is associated with expression of PCNA (Proliferating Cell Nuclear Antigen) which is a subunit of DNA polymerase delta and expresses in the replicating cells during late G1 phase to early S phase of the cell cycle (5, 6).

Estrogen exerts its biological activity through two nuclear receptors, estrogen receptor-α (ERα) and estrogen receptor-β (ERβ) (7). The estradiol-17β is the naturally occurring ligand bound with ligand binding domain (LBD) of estrogen receptors (ER). The chemical nature of phytoestrogens is attributed to its affinity for ERα and ERβ (8) and reported to be in food sources of both human and animals (9). Such compounds have been isolated and reported to act as Selective Estrogen Receptor Modulator (SERM). Genistein is a plant derived isoflavone that shows binding interaction with the ERs within the same active site as that of estradiol-17β (10). Similarly, tamoxifen is a SERM acting as agonists in the uterus and antagonist in breast tissue by interacting with estrogen receptors (11). Binding affinity with ER and docking studies of such compounds have been reported earlier. Toremifene is another compound which shows docking and binding interactions with estrogen receptors (12). Several compounds from plants sources like Dioscorea villosa and Ginkgo biloba showed remarkable docking with both ERα and ERβ or selectively with anyone of the receptors (13).

Prior to determination of compounds’ binding affinity with steroid hormone receptors and in silico docking studies, chromatographic separation and identification of the compounds from complex mixtures like crude plant extract is inevitable. Gas chromatography-mass spectroscopy (GC-MS) is a compatible technique used for the identification and quantification of the unknown organic compounds in a complex mixture by interpretation of the matching spectra with the reference spectra (14). In silico molecular docking study is one of the most efficient tools to determine the ligand-receptor binding interaction of a particular compound. In the present research, following separation of the phytocompound(s) by GC-MS, only selected compounds were considered for in silico studies with human ERα and ERβ.

The present study aimed at showing the presence and identifying the compounds showing estrogenic properties in *Scoparia dulcis*. This study can help in identifying the potential estrogen mimic or antiestrogenic compounds. The results of this investigation can be used to isolate each of the compounds identified and evaluate estrogenic property of each of the compounds.

## Methods

### The plant materials and extract preparation:

*Scoparia dulcis* was collected from Arunachal Pradesh and Assam in the north eastern region of India. The plant was identified using standard reference books and with the help of taxonomists of Rajiv Gandhi University. The aerial parts (Leaves and stem) of the plant were shade dried, ground to 60 mesh powder. Extract was prepared following method used in our laboratory (15). The dry powder was macerated in methanol (In a ratio of 1:4) and kept at room temperature with occasional stirring for 72 *hr*. The filtrate (Extract) was concentrated and dried using a rotary vacuum evaporator at a temperature of 27±2°*C*, stored at −20°*C* and was used for experiments.

### Experimental animal:

LACA strain Swiss albino mice (25±3 *g*) were used as the experimental animal. The animals were reared in the Central Animal Facilities of Rajiv Gandhi University. The animals were kept under normal environmental conditions of natural dark and light period and regulated temperature of 20–27°*C*. Animals were fed with standard pellets, fresh bengal gram, cereals and water ad libitum. Experiments were performed on cyclic ovary intact females and ovariectomized (OVX) mice models. Ovariectomy was done following standard method (16).

### *In vivo* experimental design:

The adult cyclic mice within the age group of 60–70 days were divided into two groups with 6 mice in each group. Group I: Cyclic control (Vehicle treated) and Group II: Cyclic extract treated. The ovariectomized (OVX) mice under the similar age group were divided into three groups with six mice in each group as, Group III: OVX-vehicle treated (Control), Group IV: OVX-estradiol-17β (E2) treated and Group V: OVX-extract treated. Dried methanolic crude extract of *Scoparia dulcis* was suspended in 500 *µl* distilled water to prepare a final dose of 500 *mg/kg* body weight/day and administered to each female mice during 7.00–9.00 *hr*. The cyclic ovary intact females were treated for 8 consecutive days beginning with proestrus, while the OVX females (Group V) were treated with the extract for three days in a similar manner. OVX-vehicle treated (Control) females (Group III) received the vehicle for E2 (Sesame oil) in a dose of 100 *µl/day* for three consecutive days. Estradiol-17β was injected subcutaneously at the dose of 100 *ng*/100 *µl* for 3 consecutive days to OVX females (Group IV). Both control and extract treated cyclic females were sacrificed next day (Day 9) of the last treatment (Day 8) during 7.00–9.00 *hr* of sample collection. OVX females were sacrificed on day 4 of sample collection.

### Immunohistochemistry of PCNA and signal analysis:

The evaluation of the estrogenic effect of the crude extract on the uterine tissue was carried out by localizing the proliferating cell nuclear antigen (PCNA). Detection of PCNA in uterine sections was done immunohistochemically in paraffin- embedded tissue sections following standard method (17). Briefly, uterine horns were collected and fixed in Bouin’s fixative for 72 *hr*. Tissues were washed in running tap water to remove excess fixative and dehydrated by graded series of ethanol. Ethanol was cleared in xylene and the tissues were embedded in paraffin wax. The tissues were cut in 5 *µ* thick sections in a Leica rotary microtome and sections were mounted on Poly-L-Lysine (Sigma-Aldrich) coated slides, deparaffinized, rehydrated and exposed to antigen retrieval using citrate buffer. The immunohistochemical examination was performed using mouse monoclonal primary antibody (PC-10: SC-56, Santa Cruz Biotech) and goat anti-mouse HRP conjugated secondary antibody (SC 3697, Santa Cruz Biotech) and 3, 3-diaminobenzidine (DAB) as chromogen. The slides were mounted in DPX with cover slips and photographed using Leica DM5000B microscope. Quantification of the immunohistochemical signal of PCNA was done by ImageJ software (NIH, USA) using the standard protocol. Briefly, IHC image was loaded to be analyzed in the software by pre-setting the image type in Red-Green-Blue (RGB) mode. The area of the image to be quantified was selected using the rectangular selection tool. Following selection, the intensity value of the IHC stained area was calculated by using the analyze tool of the software. Similarly, the intensity of the total area was calculated. Finally, the mean value of the intensity of the IHC stained area and total area was calculated in percentage. In the present research work, for ease of the study, the calculated values of the signal intensity were categorized into +(<20%, Less); ++(20%–50%, Moderate) and +++(>50%, High) as shown in table 1.

**Table 1.** Signal analysis of immunohistochemistry of PCNA in mice uterus using Image J software. Data are taken in triplicate (n=6)

**Sl no**	**Samples**	**Intensity of signals (%)**
**1**	CVT	++
**2**	CET	+++
**3**	OVT	+
**4**	OE2T	+++
**5**	OET	++

+ (< 20%)=Less;

++ (<50%)=Moderate;

+++ (<80%)=High

CVT- cyclic vehicle treated, CET- cyclic extract treated, OVT- OVX vehicle treated (Control), OE2T- OVX E2 treated, OET- OVX extract treated

### GC-MS analysis:

GC-MS analysis was carried out on a GC SHIMADZU QP2010 system comprising a AOC–20i auto sampler and gas chromatograph interfaced to a mass spectrometer (GC-MS) instrument employing the following conditions: elite-1 fused silica capillary column was used (330 *mm* x0.25 *mm* IDx1 *μm* df, composed of 100% dimethylpolysiloxane), operating in an electron impact mode at 70 eV; helium (99.999%) was used as carrier gas at a constant flow of 1 *ml/min* and an injection volume of 0.5 *μl* was employed (Split ratio of 10.1) at injector temperature of 250*°C* and ion-source temperature 280*°C*. The oven temperature was programmed from 110*°C* (Isothermal for 2 *min*), with an increase of 10*°C/min*, to 200*°C*, then 5*°C/min* to 280*°C*, ending with a 9 *min* isothermal at 280*°C*. Mass spectra were taken at 70 eV with a scan interval of 0.5 *s* and fragments from 40 to 550 *Da*.

### In silico docking studies of selected phytocompounds against ERα and ERβ:

The identified compounds from GC-MS analysis were used for energy minimization using 2000 steps of steepest descent followed by 5000 steps of conjugate gradient for geometry optimization in Impact module of Schrodinger 2014. The crystal structures of ERα (PDB ID: 4PP6) and ERβ (PDB ID: 3OMQ) were retrieved from Research Collaboratory for Structural Bioinformatics (RCSB) databank. The receptor structures were prepared using protein preparation wizard where the addition of hydrogen atoms and correction of bond-orders followed by energy minimization in steepest descent optimization technique were carried out. Finally, ERα and ERβ receptors and energy minimized compounds were employed for understanding the binding mode using Induced Fit Docking where the active site of the protein (>20Ang) and ligand were kept flexible (18). For all computational study, Optimized Potentials for Liquid Simulations (OPLS) force-field was used for describing energy profile of molecules. For comparative binding studies of these compounds, Glide -XP refine mode docking of co-crystal compounds has been also carried out. Topmost binding mode of these compounds was also analyzed using docking score and glide energy.

### Molecular dynamics simulation (MDS):

Molecular dynamics (MD) simulations of the protein-ligand complexes were carried out to investigate the dynamic behavior properties. These were performed in Gromacs 2018.3 (19, 20). The gromacs force field (GROMOS4a31) and general amber force field (GAFF) were used to generate the parameters for the protein (21–23). The ligand topology files were generated using the PRODRG server (24). Complex systems of protein-ligand built a dodecahedron box and it was solvated with three-point water model (SPC water). To neutralize the charge of the system, Na+ ions were added to the box as counter ions. To preserve the systems in stable environment (300 *K*, 1 *Bar*), Berendsen temperature and pressure coupling methods were used, and the coupling constants were set to 0.1 *nm*. Each complex was minimized via steepest descent algorithm for 1 *ps*, then a restrained molecular dynamics simulation was conducted for 20 *ps* to equilibrate the water around the system and make it equal. Particle Mesh Ewald (PME) was applied for calculations of the long-range electrostatics. All bond lengths were restrained using the Linear Constraint Solver (LINCS) algorithm (25). Finally, the position restraints were released and 100 *ns* production MD simulation was performed.

### Ethical approval:

Animal usage and the protocols were approved by the Institutional Animal Ethics Committee of Rajiv Gandhi University, Itanagar, Arunachal Pradesh, India (IEAC/RGU/17/16;IAEC/CPCSEA).

## Results

### PCNA immunohistochemistry:

**Figure 1.**
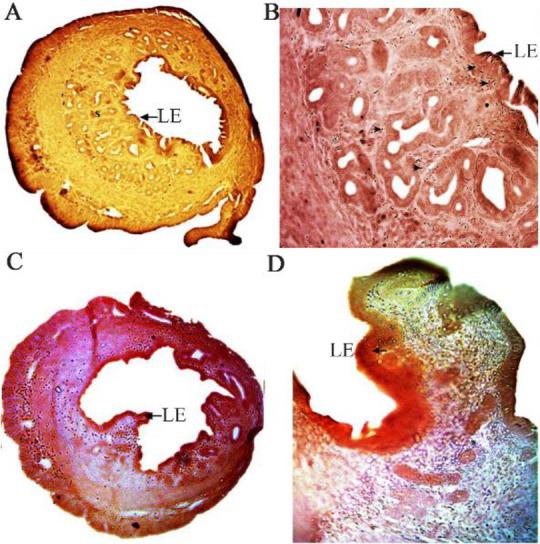
Immunohistochemistry of PCNA in cyclic control (A & B) and cyclic extract treated (C & D) mice. PCNA expression (arrow head) is found in the endometrial stromal cells (S) and luminal epithelium (LE). Original magnification: A & C −5X: B & D −40X

**Figure 2.**
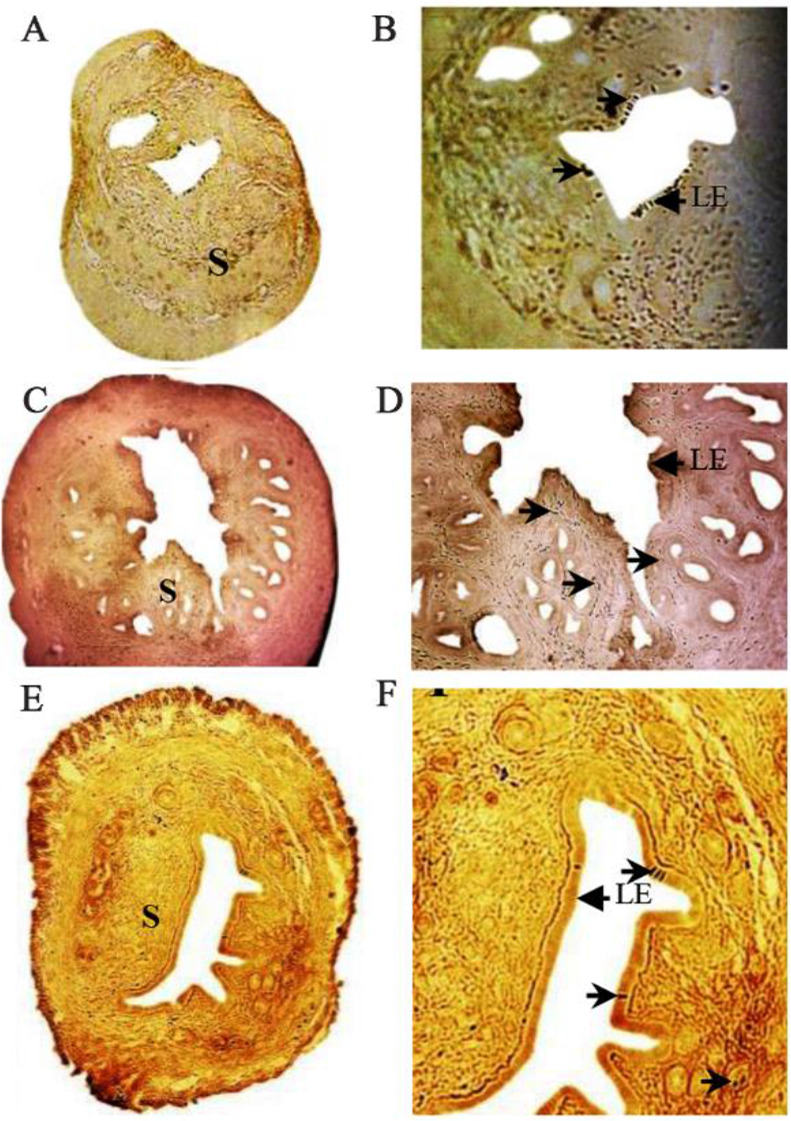
Immunohistochemistry of PCNA in OVX-vehicle treated (control) (A & B), OVX-E2 treated (C & D) and OVX- extract treated (E & F) mice. PCNA expression (arrow head) is found in the endometrial stromal cells (S) and luminal epithelium (LE). Original magnification: A, C & E −5X: B, D & F −40X

The effect of plant extract on uterine histoarchitecture was studied using immunohistochemical staining of PCNA presented in figures 1 and 2. In the cyclic control uterus, the endometrial epithelium was intact with the uniform layer of epithelial cells without any significant proliferation (Figures 1A and B). The cyclic extract treated mice showed extensive proliferation of the endometrial epithelium (Figures 1C and D). The endometrial stromal cells also showed more expression of PCNA in comparison to the cyclic control. The signal analysis in table 1 also confirmed strong immunostaining of PCNA in cyclic extract treated mice (+++). The OVX-vehicle treated (Control) rats showed no proliferation of the endometrial epithelium (Figures 2A and 2B), and expression of PCNA was also less in the stromal tissues (+). In the OVX- E2 treated mice (Figures 2C and 2D), the PCNA was expressed in the endometrial stromal cells with high intensity. The OVX-E2 treated mice showed strong immunostaining (+++). The OVX-extract treated mice showed expression of PCNA in luminal epithelium and in endometrial cells (Figures 2E and 2F). The OVX females treated with extract showed moderate immunostaining of PCNA in uterine epithelium (++).

### GC-MS analysis of compounds and their in silico molecular docking studies against estrogen receptors:

**Figure 3.**
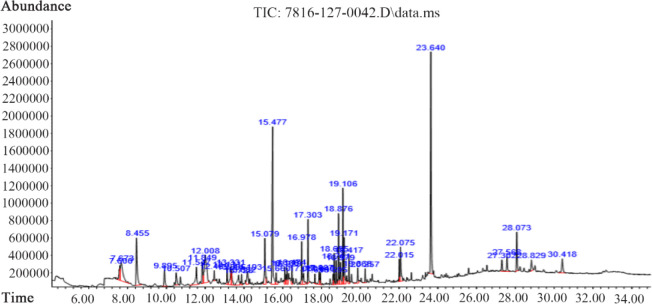
GC-MS chromatogram of methanolic extract of *scoparia dulcis*

The results pertaining to GC-MS analysis led to the identification of a number of compounds from the GC fractions of the plant extract (Figure 3). These compounds were identified by Mass spectrometry attached to GC. Initially, docking studies of 45 phytochemical compounds were carried out against both estrogen receptor α and β. However, only seven (Probability/Qual ≥70%) compounds showed binding interaction with human ERα and ERβ. These compounds with their PubChem ID, retention time (RT), concentration/area (%) and their probability matching with National Institute of Standards and Technology (NIST) library are listed in table 2. This table also contains the information regarding, whether these identified compounds have been previously reported from Scoparia genus or/and any other plants along with the references, if any.

**Table 2.** Identified compounds from methanolic extract of *scoparia dulcis* linn

**Sl. no**	**Pubchem ID**	**RT**	**Area (%)**	**Compound**	**Previous report on the presence of the identified compound in scoparia genus**	**Previous report on the presence of the identified compound on any plant**	**Probability/Qual (%)**
**1**	10329	8.455	5.13	2,3-dihydro-benzofuran (Coumaran)	No	Yes (26, 27, 28)	87
**2**	243	7.601	1.29	Benzoic acid	No	Yes (29, 30)	96
**3**	10772	15.475	11.15	6-methoxy-2-benzoxazoline (Coixol/MBOA)	Yes (31, 32)	Yes (31)	97
**4**	5366075	13.729	1.53	3-hydroxy-.beta.-damascone	No	Yes (33)	70
**5**	6437599	13.328	1.84	Megastigmatrienone	No	Yes (34)	89
**6**	8201	18.877	3.41	Methyl stearate	Yes (35)	Yes (36, 37)	99
**7**	445639	19.174	2.58	Octadecanoic acid (Stearic acid)	Yes (38)	Yes (39)	99

### In silico molecular docking studies of phytocompounds against estrogen receptors:

**Figure 4.**
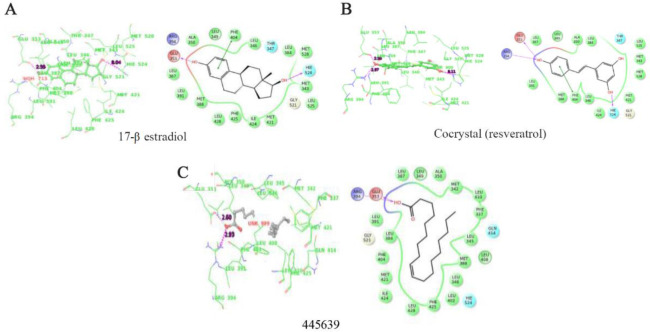
A–C) Binding interaction of estradiol-17β, A) and cocrystal (resveratrol) B) and Octadecanoic acid, C) from the *Scoparia dulcis* extract which showed highest binding affinity among the seven docked compounds with ERα

**Figure 5.**
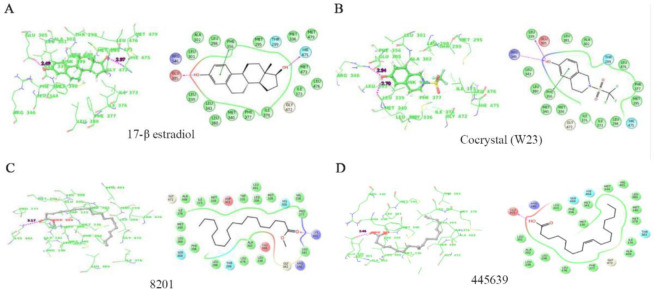
A–D) Binding interaction of estradiol-17β, A) and cocrystal (2-[(trifluoromethyl) sulfonyl]-1,2,3,4-tetrahydroisoquinolin-6-ol), B) and two compounds methyl stearate, C) and Octadecanoic acid, D) from *Scoparia dulcis* extract which showed highest binding affinity among the seven docked compounds with ERβ

The docking studies of the compounds with ERα and ERβ are presented in figure 4 and figure 5. Based on docking score, glide energy and binding interaction with the active site, best compounds were short listed. Binding affinity in terms of score and energy for the best compounds with ERα and ERβ is tabulated in table 3 and table 4.

**Table 3.** Docking results of selected phytocompounds against estrogen receptor α (PDB ID: 4PP6)

**Compound (PubChem ID)**	**Docking score (*kcal/mol*)**	**Glide energy (*kcal/mol*)**	**Hydrogen bond interactions**	**Distance (Å) D-H...A**
**Octadecanoic acid (445639)**	−6.15	−46.92	UNK 999 (O)… N ARG (394) UNK 999 (O)… O GLU (353)	2.9 2.6
**Benzoic acid (243)**	−6.50	−32.20	UNK 999(O)…N ARG(394) UNK 999(O)…O GLU(353) UNK 999(O)… O LEU(346)	3.1 2.5 2.7
**Cocrystal (resveratrol)**	−9.44	−34.92	UNK 999 (O) …N ARG (394) UNK 999 (O) …O GLU(353)	2.7 2.6
**Estradiol-17β**	−11.57	−53.89	UNK999 (O)...N His524 UNK999 (O)...O Glu353	3.2 2.5

**Table 4.** Docking results of selected phytocompounds against estrogen receptor β (PDB ID: 3OMQ)

**Compound (PubChem ID)**	**Docking score (*kcal/mol*)**	**Glide energy (*kcal/mol*)**	**Hydrogen bond interactions**	**Distance (Å) D-H...A**
**Methyl stearate (8201)**	−8.85	−49.59	LYS 401 (N)… OUNK 999	3.1
**Octadecanoic acid (445639)**	−8.73	−49.49	UNK 999 (O)… O GLU (305)	2.4
**3-hydroxy-.beta.-damascone (5366075)**	−7.49	−34.82	UNK 999 (O)…O GLU (305)	2.9
**6-methoxy-2-benzoxazoline (10772)**	−5.01	−26.95	UNK (ð)… (ð) PHE(356) UNK 999 (N)…O LEU (339)	3.1
**Benzoic acid (243)**	−6.10	−29.07	UNK 999 (O)…O LUE (339) UNK 999 (O)…O GLU (305) ARG 346 (N)…O UNK (999)	3.0 2.4 2.8
**Cocrystal 2-[(trifluoromethyl)sulfonyl]-1,2,3,4-tetrahydroisoquinolin-6-ol**	−7.52	−41.62	UNK 999 O…O LEU (339) UNK (ð)… (ð) PHE(356)	2.9
**Estradiol-17β**	−11.85	−52.33	UNK (O)...O Glu305 UNK (O)...N His475	2.4 2.9

Estrogen (estradiol-17β) interacts with Glu353 (H3), Arg394 (H5) and His524 (H11) by hydrogen bonds. The structural arrangement of helices is very well organized in agonist bound ERα. Resveratrol, which is a phytophenolic compound, has been reported for estrogen modulating activity similar to the estradiol-17β. From the docking studies with ERα, Octadecanoic acid with PubChem ID 445639 showed favorable glide energy compared to the cocrystals of resveratrol (Table 3). This compound also has similar hydrogen bond interactions with Glu353 and Arg394 which are also observed in case of substrate and resveratrol binding. The hydrophobic interactions of this compound with residues His524, Met388, Phe404 are also similar (Figure 4). Docking studies with ERβ showed compounds methyl stearate, Octadecanoic acid with PubChem IDs 8201, 445639, respectively to have good docking score as well as glide energy in comparison of cocrystal (Table 4). This table also lists other three compounds which don’t have comparable glide energy with the cocrystal and estradiol-17β. These compounds have hydrogen bonds with Lys401 and Glu305, respectively. They have hydrophobic interactions with active site residue such as Leu343, Leu380, Met-340, Met336, and Gly472 which are consistent with the substrate and cocrystal ligand (2-[(tri-fluoromethyl) sulfonyl]-1,2,3,4-tetrahydroisoquin-olin-6-ol). In spite of low energy and score, the compounds such as 6-Methoxy-2-benzoxazoline, PubChem ID: 10772 showed both hydrogen bonding with Leu339 and π-π interactions with Phe356 as observed in the cocrystal ligand (2-[(trifluo-romethyl) sulfonyl]-1,2,3,4-tetrahydroisoquinolin-6-ol).

### Molecular dynamics simulation:

**Figure 6.**
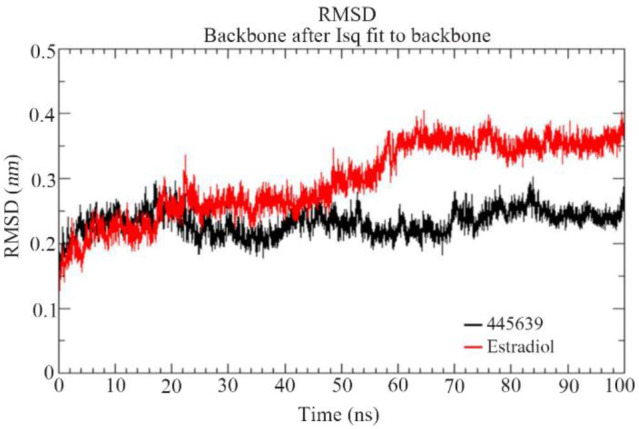
RMSD graph of the best compound octadecanoic acid (445639) and estradiol docked complexes of estrogen receptor alpha (PDBID: 4PP6) from the MD trajectories

**Figure 7.**
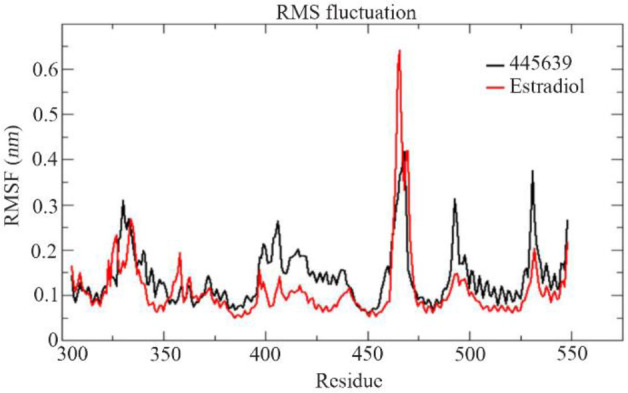
RMSF graph of the best compound octadecanoic acid (445639) and estradiol docked complexes of estrogen receptor alpha (PDBID: 4PP6) from the MD trajectories

**Figure 8.**
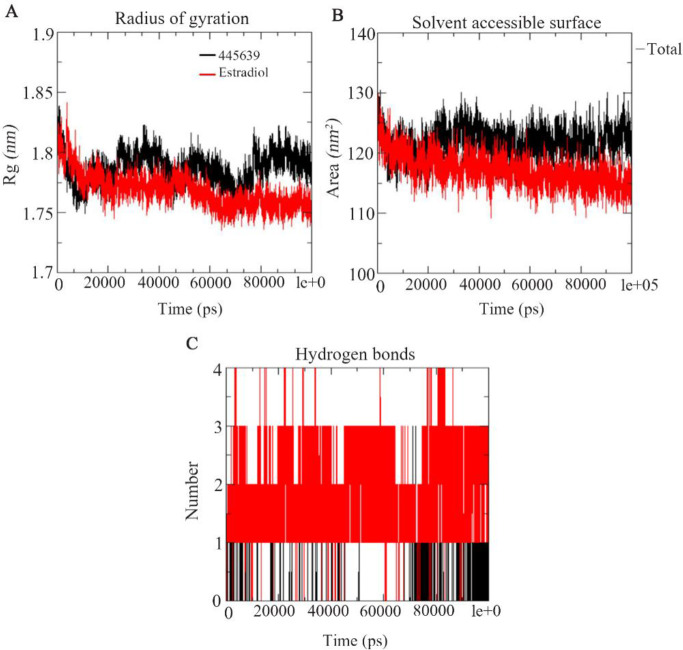
The best compound octadecanoic acid (445639) and estradiol docked complexes of estrogen receptor alpha from the MD trajectories. A) Radius of gyration, B) Solvent accessible surface area, C) Hydrogen bond contacts

Based on the induced docking results of both estrogen receptor α and β, the best compound Octadecanoic acid (PubChem ID: 445639) and standard drug of estradiol-17β were chosen to understand their stability and conformational changes using 100-*ns* time span molecular dynamics simulation Further, an attempt was made to study Root-Mean-Square Deviation (RMSD), Root-Mean-Square Fluctuation (RMSF), Radius of Gyration (Rg), Solvent-Accessible Surface Area (SASA) and hydrogen bond analysis between the best compound and standard drug (Estradiol) of ERα (Figures 6, 7 and figure 8) protein structures. In the MD results of estrogen receptor α, the overall RMSD of C-α atoms from the identified compound Octadecanoic acid (PubChem ID: 445639) shows the stable convergence throughout the simulation period. It depicts RMSD values of 0.22–0.29 *nm*, respectively. Estradiol docked complex shows the higher deviations (0.2 *nm*) from the initial 0 to 30 *ns* time period. Later, it gradually acquired convergence after 50 *ns*. It maintained the RMSD of 0.27 *nm*, after the relaxation period of 50 *ns*, to produce stable trajectories in simulation. The backbone RMSD values reach the maximum (0.40 *nm*) till the end of simulation (Figure 6).

To predict the dynamic behavior of the residual fluctuation for docked complexes throughout the simulation, for the best compound Octadecanoic acid (PubChem ID: 445639) and estradiol, RMSF values were calculated and are shown in figure 7. RMSF analysis reveals the higher flexibility of residual fluctuation in the best compound than estradiol protein complexes. Radius of gyration determines the mass-weight of collection of atoms and their common center of mass. Rgyr was plotted based on the Cα atoms of the protein. In figure 8, the best compound, Octadecanoic acid (445639), showed good Rg value at the end of the simulation compared with estradiol; the compound Rg value was 1.84 *nm* at 0 *ps*, 1.78 *nm* at 70000 *ps* and finally at the end of the simulation, it was 1.79 *nm* at 100000 *ps*. Estradiol at 0 *ps* showed similar value of the best compound which was 1.84 *nm*; afterwards, it gradually decreased throughout the simulation. At the 60000 to 100000 *ps*, the value turned out to be 1.75 *nm* (Figure 8A). Solvent-accessible surface area was analyzed for describing bimolecular surface area and how much access is available to the solvent molecule. From figure 8B, it is clear that the best compound showed no deviation at the overall MD trajectories, but when compared to estradiol, it had significantly lesser deviations. SASA values were 124.5 Å and 121.4 Å to 115.5 Å, respectively. In addition to that, total number of hydrogen bond contacts was also analyzed. Figure 7C depicts that estradiol has the maximum of four hydrogen bond interactions throughout the simulation. The best compound showed only one hydrogen bond interaction from initial 0 *ns* till the end of the 100 *ns* simulation MD trajectories.

## Discussion

During the present investigation, it was found that the methanolic crude extract of *Scoparia dulis* induces the proliferation of the endometrial epithelium in mice uterus in presence or absence of ovary *in situ*. This induced cell proliferation could be due to the agonistic effects of the compound(s) present in the crude extract of the plant. The plant-derived compounds called phytoestrogens have the capability of binding to estrogen receptor (ER) and exert various estrogenic and antiestrogenic effects. Phytoestrogens are often used as the natural alternative to hormone replacement therapy (HRT) and to reduce menopausal symptoms as well as reduce other female reproductive abnormalities (40, 41). Many of these phytoestrogens like resveratrol and trans-resveratrol demonstrate a broad spectrum of pharmacological and therapeutic health benefits (42, 43). The uterotrophic property of coumestrol and genistein and many other phytoestrogens’ effects on estrous cycle, oocyte maturation, fertilization and sequential embryonic development have been reported earlier (44, 45).

Our present in silico molecular docking studies showed that phytocompounds [Octadecanoic acid (PubChem ID: 44539), and methyl stearate (PubChem ID: 8201)] present in the methanolic extract of *Scoparia dulcis* bind with ERα and ERβ. Out of four compounds, (PubChem ID: 44539) showed maximum binding interaction with both receptors. Another compound (PubChem ID: 8201) showed binding interaction with only ERβ. The glide energy of this compound is higher than the other two compounds which also showed interaction with the ERβ. Phytocompounds’ binding affinity with estrogen receptors has been reported earlier in other plants (46). Estrogen is considered as one of the primary requirements and crucial for endometrial receptivity as well as successful pregnancy (47). In our present study, compounds mentioned above showed higher binding interaction in terms of glide energy with ERα and ERβ than that of respective cocrystals (Resveratrol) and 2-[(trifluo-romethyl) sulfonyl]-1,2,3,4-tetrahydroisoquinolin-6-ol]. These compounds’ binding interaction with both the receptors is close to that of estradiol-17β. In uterine tissue, estradiol-17β is known to stimulate physiological binding with ERα only (48). Estrogenically active compounds in other plants have been reported earlier (49).

Several studies have been done on various phytocompounds present in plants used in reproduction regulation showing interaction with estrogen receptors. Presence of phytoestrogens in extract of plants like Vitex agnus-castus and Glycyrrhiza glabra reported docking score showing strong docking ligands of estrogen receptors (50) in comparison to existing cocrystals. These compounds showed binding with the estrogen receptors and also showed estrogenic activity (49, 51–53). These reflect that the docking interactions results can be collated with the estrogenic (agonist or antagonist) activity of the compounds. The present study also manifested that the extract has estrogenic activity *in vivo* and on the basis of the docking score of the selective compounds being higher than the existing cocrystals. These compounds can also be suggested to be possible estrogen agonistics. Effects of compounds from *Scoparia dulcis* on other physiological aspects have also been reported (54). However, estrogenic effects of *Scoparia dulcis* and binding interaction with human estrogen receptors have not been reported earlier.

## Conclusion

In our present research, compounds in *Scoparia dulcis* with estrogenic properties have been reported. This effect was shown for the first time through *in vivo* test in animal model system and in silico studies with human estrogen receptors. It has been speculated that these compounds exert agonistic effects of estradiol-17β in the uterine endometrium evidenced by the expression of PCNA. These compounds could be used as potential selective estrogen receptor modulators for pharmacological purposes especially for reproduction regulation. This study can be considered as the starting point leading to isolation of each of the compounds from *Scoparia dulcis* which show major interactions with the ERs and they can be tested individually for their potential estrogenic activities through optimal experimental design in near future.
